# OSTEOCHONDRITIS DISSECANS OF THE HIP IN LEGG-CALVÉ-PERTHES DISEASE: CASE REPORT AND REVIEW

**DOI:** 10.1590/1413-785220243201e277177

**Published:** 2024-03-22

**Authors:** Luiz Renato Agrizzi de Angeli, Bárbara Lívia Corrêa Serafim, Felippi Guizardi Cordeiro, Felipe Spinelli Bessa, Daniel Augusto Carvalho Maranho

**Affiliations:** 1 Universidade de São Paulo, Hospital das Clínicas da Faculdade de Medicina, Institute of Orthopedics and Traumatology, São Paulo, SP, Brazil.; 2 Hospital Israelita Albert Einstein, São Paulo, SP, Brazil.; 3 Núcleo de Ortopedia Especializada de São Paulo, São Paulo, SP, Brazil.; 4 Hospital Sírio Libanês de Brasília, Orthopedics Group, Brasília, GO, Brazil.

**Keywords:** Orthopedics, Orthopedic Procedures, Growth And Development, Growth Plate, Legg-Calve-Perthes Disease, Osteochondritis Dissecans, Ortopedia, Procedimentos Ortopédicos, Crescimento e Desenvolvimento, Lâmina de Crescimento, Doença de Legg-Calve-Perthes, Osteocondrite Dissecante

## Abstract

**Introduction:**

Legg-Calvé-Perthes disease (LCPD) is the idiopathic osteonecrosis of the capital femoral epiphysis in children. It is a self-healing condition, and the morphology of the hip may vary according to the severity of the disease, among several other factors. The treatment focuses on attempts to prevent femoral head collapse, obtain functional hip motion recovery, and reduce pain. Osteochondritis Dissecans (OCD) of the femoral head has been reported in 2% to 7% of patients diagnosed with healed LCPD. Although OCD may remain asymptomatic, the osteochondral fragment has the potential to become unstable, evolving into symptoms of pain, locking, catching, and snapping.

**Case report:**

We present a case report of a ten-year-old boy with an OCD lesion following LCPD who underwent effective osteochondral fixation through the surgical hip dislocation approach. The patient evolved to excellent functional recovery at 1 year post-operatively.

**Discussion:**

The surgical hip dislocation approach allows anatomical fixation of the OCD fragment, as well as improvement of hip biomechanics, decreasing pain, improving range of motion and joint congruency, and preserving the native articular cartilage. It also gives the surgeon the opportunity to assess hip stability, femoroacetabular impingement and labral tears, allowing a wide variety of options for the treatment of the healed LCPD. ***Level of Evidence IV; Type of study Case Report.***

## RESUMO

**Introdução:**

A Doença de Legg-Calvé-Perthes (DLCP) é a osteonecrose idiopática da epífise femoral proximal em crianças. É uma condição auto resolutiva, porém a morfologia final do quadril pode variar de acordo com a gravidade da doença. O tratamento concentra-se na tentativa de prevenir o colapso da cabeça femoral, obtendo recuperação funcional do movimento do quadril e redução da dor. A osteocondrite dissecante (OCD) da cabeça femoral foi relatada em 2% a 7% dos pacientes diagnosticados com DLCP já curada. Embora a OCD possa permanecer assintomática, o fragmento osteocondral tem potencial para se tornar instável, evoluindo para sintomas de dor, bloqueio, impacto e estalido.

**Relato de caso:**

Apresentamos o relato de caso de um menino de 10 anos com OCD da cabeça femoral após DLCP, submetido à fixação osteocondral do fragmento por meio da abordagem cirúrgica de luxação do quadril. O paciente evoluiu com excelente recuperação funcional 1 ano após a cirurgia.

**Discussão:**

A abordagem cirúrgica da luxação do quadril permite a fixação anatômica do fragmento da OCD, bem como a melhora da biomecânica do quadril, diminuindo a dor, melhorando a amplitude de movimento e a congruência articular e preservando a cartilagem articular nativa. Também dá ao cirurgião a oportunidade de avaliar a estabilidade do quadril, impacto femoroacetabular e lesões labrais, permitindo uma ampla variedade de opções para o tratamento das sequelas da DLCP. ***Nível de evidência IV; tipo de estudo Relato de Casos***.

## INTRODUCTION

Legg-Calvé-Perthes disease (LCPD) refers to idiopathic osteonecrosis of the capital femoral epiphysis in children.^1–4^ It is a self-healing condition in which the blood supply to the capital femoral epiphysis spontaneously recovers by means of recanalization of pre-existing vessels that occurs precociously within weeks after the necrosis, or eventually formation of new vessels over a period of months to years.^1,4–7^ The clinical onset of LCPD usually occurs in children between ages of four and eight years, with the condition being five times more common in males than females.^1,2,5^ During the healing process, abnormal proximal femoral growth results in varying deformities, which predisposes to hip joint incongruity, femoroacetabular impingement, chondrolysis and labral tears.^8–11^

The clinical management of Perthes’ disease focuses on attempts of preventing femoral head collapse, obtaining functional recovery of the hip motion and reduction of pain.^2,8,12^ The prognosis of the hip joint affected by LCPD depends on the age of the patient at the time of onset, patient weight, the stage of the disease, the extent of epiphyseal involvement, height of the lateral pillar and the extrusion of the femoral head.^5,8,10,13^

Osteochondritis Dissecans (OCD) of the femoral head has been reported in 2% to 7% of patients diagnosed with LCP disease.^9,14^

These lesions often remain asymptomatic, although the osteochondral fragment may become unstable and release articular loose bodies, producing symptoms of pain, locking, catching and snapping.^2,9,12,14^ Patients with osteochondral lesions of the femoral head are at risk of rapid progression to symptomatic osteoarthritis of the hip joint.^15–17^ The surgical hip dislocation approach has been used to assess both intra-articular and extra-articular abnormalities around the hip joint as it is capable of addressing residual deformities of the proximal femur in patients with healed LCPD.^6,8–11^

In this case report, we present a patient with an OCD lesion following Perthes disease treated through a surgical hip dislocation approach. It was possible to effectively treat the OCD lesions and preserve the native articular cartilage, avoiding alternative cartilage substitution techniques as salvage procedures.

This report was structured according to the CAse REport (CARE) guidelines checklist, in order to capture useful clinical information and key components from the case.^18^

### Case report

We present the case of a boy who developed a right-sided limp at the age of ten (April, 2017). He was diagnosed with LCPD and underwent conservative treatment with physiotherapy, and weight bearing restrictions using a wheelchair followed by two crutches during 28 months. At the reossification stage (IIIB) of the disease, the boy was asymptomatic, although the radiographs showed an abnormal hip morphology with flattening of the femoral head, coxa magna and brevis. Four years after the beginning of the symptoms (June, 2021, at the age of fourteen), he presented with hip pain, limping and a limited range of motion of the right hip (flexion up to 90 degrees, internal rotation up to 15 degrees, abduction up to 15 degrees).

**Figure 1:**
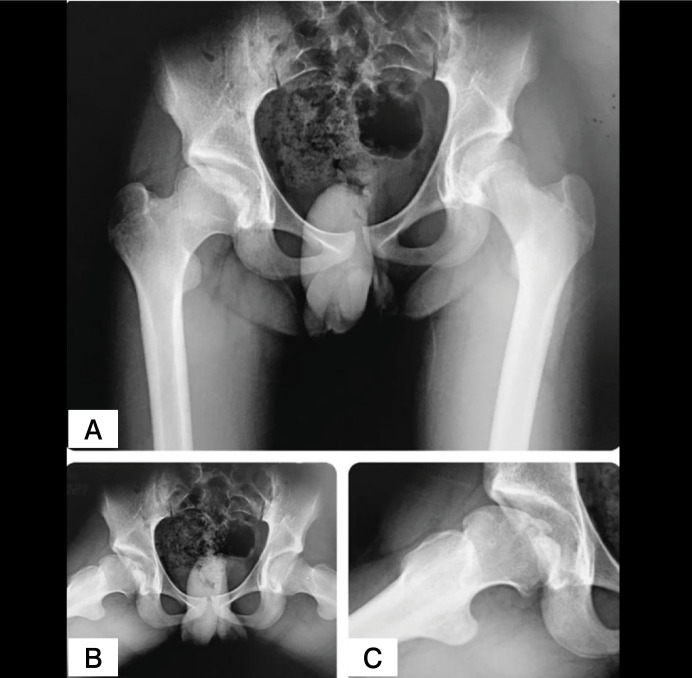
Pelvis AP (A), Frog-leg (B) and close view (C) of the right hip in the Frog-leg view radiographs. Note an osteochondral fragment in the central part of the femoral head. In the Frog-leg view, the fragment appears to be detached from its bony base.

**Figure 2:**
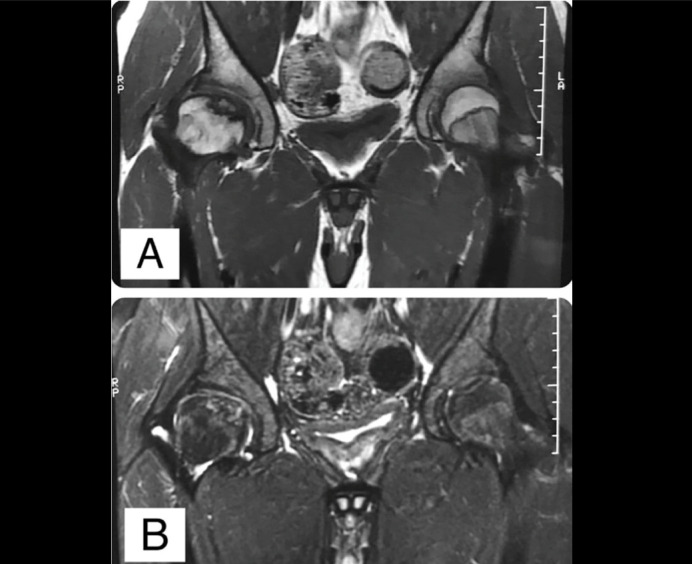
Magnetic Resonance Images (MRI) of both hips in the coronal view. A. T1-weighted MRI showing a non-perfused area surrounding the osteochondral fragment. B. T2-weighted MRI showing intra articular synovitis and edema around the OCD fragment. Note that the articular cartilage has a step-deformity due to a slight elevation of the unstable OCD fragment.

**Figure 3:**
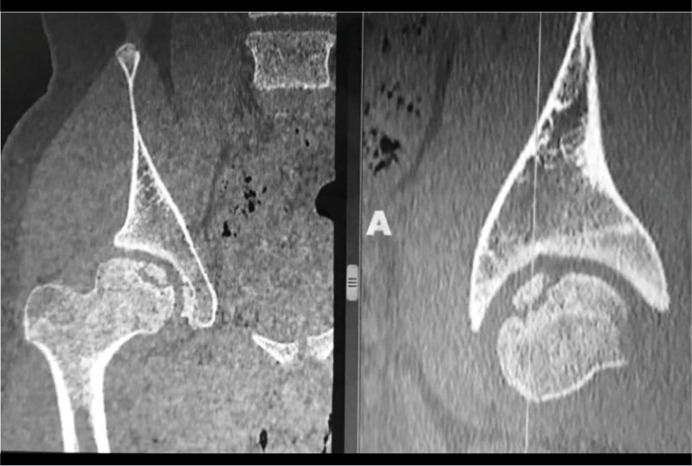
Computed tomography (CT) scan in the coronal (left) and axial (right) views. Note the elevation of the osteochondral (OCD) fragment in both views. There is no visible bone connection between the OCD fragment and the remaining of the femoral head.

Radiographs showed a possible loose osteochondral fragment inside the joint, detached from the central part of the femoral head (Figure 1). The contrast-enhanced magnetic resonance imaging (MRI) showed an unstable osteochondral fragment measuring 28 x 22 mm with surrounding bone edema (Figure 2). In addition, flattening of the femoral head, shortening of the femoral neck and synovitis were noted. The computed tomography (CT) scan evidenced an unstable osteochondral fragment with similar proportions as shown by the MRI. (Figure 3)

Conservative treatment for the OCD was instituted with partial weight bearing, non-steroidal anti-inflammatory drugs (NSAIDs) and physiotherapy for eight weeks with no improvement. Therefore, open reduction and internal fixation of the osteochondral fragment was indicated. At this time point, his Harris Hip Score (HHS)^19^ was 64,325.

### Surgical Technique

**Figure 4:**
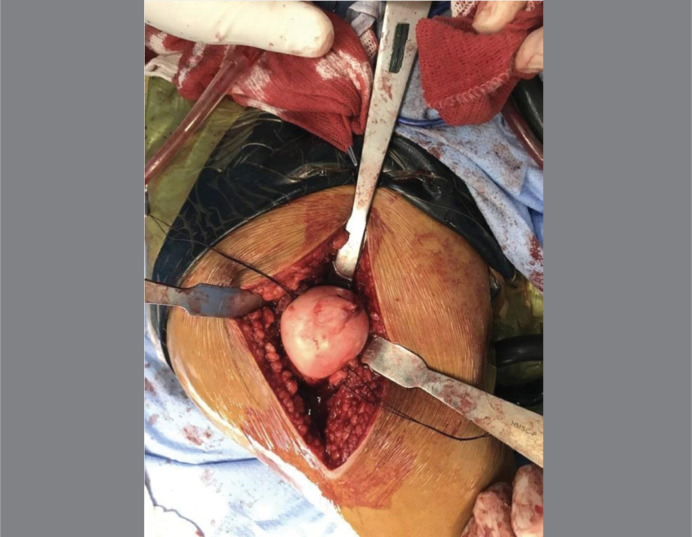
Femoral head after surgical hip dislocation. Note the ovoid shaped head and the limits of the central unstable Osteochondral Fragment. The osteochondral fragment is connected with the stable cartilage tissue.

The patient underwent OCD fragment fixation using the surgical dislocation approach as described by Ganz et al.^11^ The patient was positioned in left lateral decubitus stabilized with cushions. An incision was made over the greater trochanter in its central axis, going from 30 mm above its tip to 50 mm below its bottom part. The approach was carried through the subcutaneous tissue, sectioning the fascia lata and blunch division of gluteus maximus. The bursa was divided. The piriform tendon was identified at its trochanteric insertion. A straight trigastric osteotomy was performed at the greater trochanter with an oscillating saw, preserving the attachments of the vastus lateralis, gluteus medius and minimus. The greater trochanter was retracted anteriorly and the capsule was exposed. A “Z” shaped capsulotomy was performed, the ligamentum teres was sectioned and the hip was successfully dislocated. (Figure 4)

**Figure 5:**
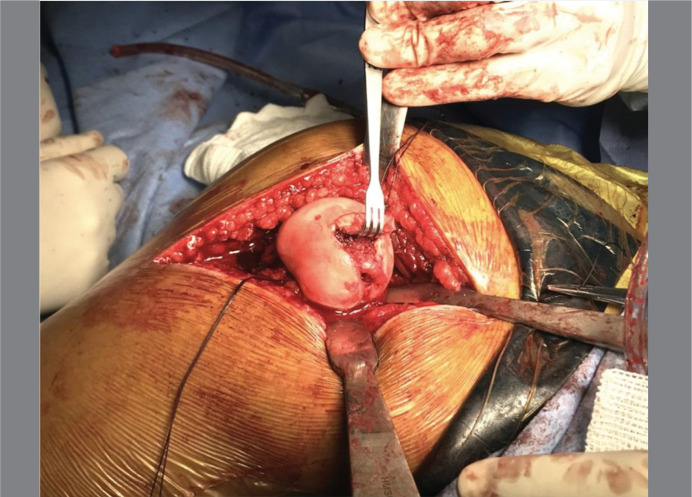
Picture showing the Osteochondral Fragment detached from its bony base after dissection with a Freer elevator, preserving the cartilage bridge connection.

**Figure 6:**
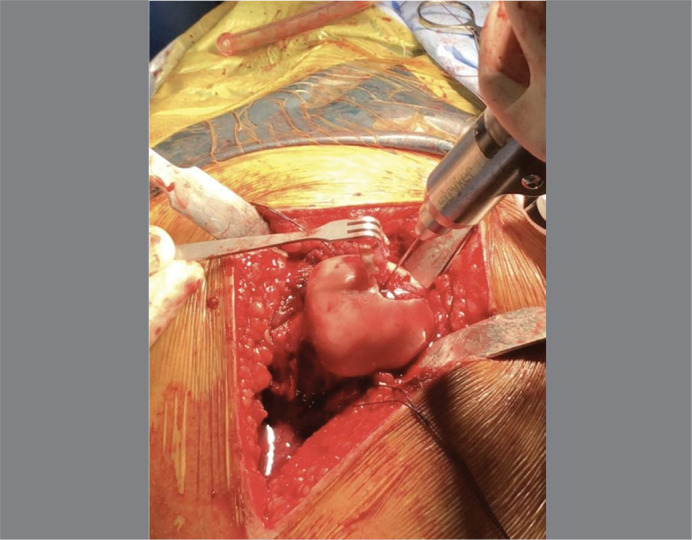
Multiple micro perforations were carried out using a 0.88 mm guidewire. Note the presence of blood at the deepest part of the lesion, showing adequate blood supply coming from the metaphysis for the healing of the Osteochondral Fragment after fixation.

**Figure 7:**
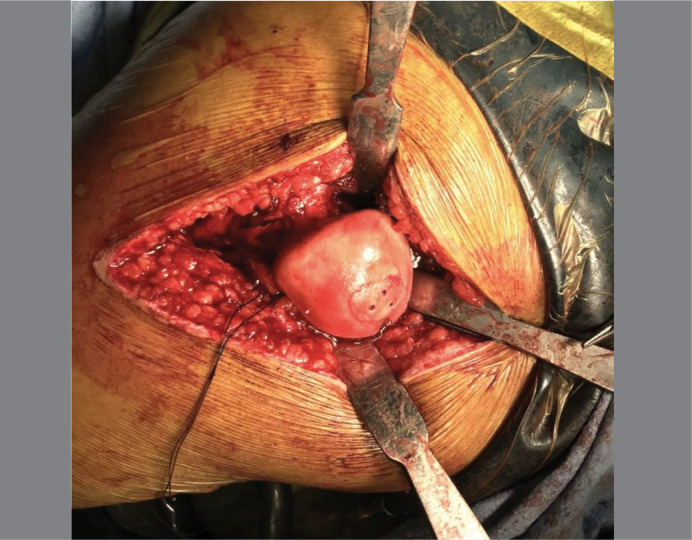
Final clinical picture after fixation of the Osteochondral Fragment with three 1.5 mm cannulated headless compression screws (Acutrak; Acumed, Hillsboro, OR). The fixation was stable and the borders of the lesion were smooth, demonstrating a good reduction.

The second part of the procedure involved the treatment of the OCD lesion. (Figure 5) First, the osteochondral fragment was gently freed from its bony base using a Freer elevator, then the underlying bony bed was debrided using a curette and multiple micro perforations were made using a 0.88 mm guidewire in order to enhance bleeding and facilitate anatomic reduction and healing of the osteochondral fragment (Figure 6). The fragment was temporarily secured using guidewires and then fixed definitively with three 1.5 mm cannulated headless compression screws (Acutrak; Acumed, Hillsboro, OR) below the surface of the joint (Figure 7). Anatomic reduction and stability were visually assessed and confirmed radiographically after the fixation. There were no labral tears or impingement following reduction of the femoral head into the acetabulum.

The capsule was loosely repaired with absorbable sutures. The greater trochanter was advanced about 1 cm and fixed with two 6.5 mm partially threaded cannulated screws with washers. The wound was closed in a standard fashion and the patient was immobilized with an abduction cushion after the procedure.

### Post Operative Care

The patient was discharged from the hospital 1 day after the procedure, immobilized in an abduction wedge cushion. Physiotherapy was initiated immediately with passive range of motion of the right hip, with exception of adduction and external rotation to protect against unwanted capsule suture failure and joint dislocation. Sitting was allowed as tolerated and no weight bearing was allowed for 8 weeks.

**Figure 8:**
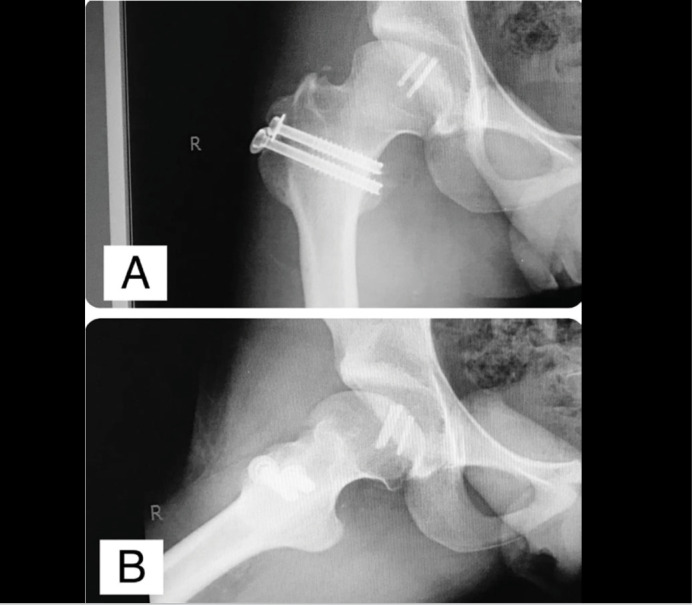
AP view (8A) and Frog-leg view (8B) of the right hip, in the 10th month postoperative. There's complete healing of the Osteochondral Fragment. The great trochanter was also healed and distalized. The implants did not bother the patient and were not removed.

The patient was seen in a 2 week interval during the first 8 weeks. After that, the patient was seen monthly until the 6th month postoperative. Radiographs were taken in each one of these visits. After great trochanter healing at the 6th week after surgery, active range of motion exercises and hip external rotation were initiated. The patient began partial weight bearing around the 8th week and gradually transitioned to full weight bearing until the 12th week. The OCD fragment healing was visible at 12th week postoperative. The physiotherapy program was continued through the first 6 months postoperative and no impact sports, running or jumping were allowed during this period. After the 6th month postoperative the patient was allowed to resume his physical activities as he used to practice before the onset of symptoms. (Figure 8)

At the latest follow-up visit 1 year postoperative, the patient presented improvement of the range of motion of the right hip (flexion up to 110 degrees, internal rotation up to 30 degrees, abduction up to 30 degrees), and was asymptomatic. His Harris Hip Score (HHS)^19^ at the final follow-up improved from 64,325 to 97, and the Trendelenburg test improved from positive to leveled.

### Patient Perspective

To evaluate the patient’s quality of life, the Harris Hip Score (HHS)^19^ was applied before surgical treatment and after complete rehabilitation. It was assessed pain, function, deformity and mobility. At 1 year postoperative, he scored a total of 97 out of 100 with a leveled Trendelenburg gait. The patient reported no pain, no difficulty in walking, being able to walk unlimited distance and to go up and down stairs without support. He also reported being able to put on his shoes without difficulty and to sit comfortably in a chair for an hour.

## DISCUSSION

LCPD is characterized by unilateral or bilateral necrosis of the femoral head, which results from a proximal femoral epiphysis ischaemia of unknown etiology and affects the range of motion of the hip.^1–4,6,7,9^ Age at disease onset and diagnosis, sex, range of motion of the hip and severity of the disease / necrosis are considered potential prognostic factors in LCPD. Despite early treatment efforts, many patients evolve with residual femoral head deformity that may be symptomatic with a residual limp and decreased hip motion^1–3,10,12,13^

OCD is rare as an isolated cause of late pain following LCPD. It has been reported to occur in 2% to 7% of the patients that present with LCPD.^9,14^ These lesions often remain asymptomatic and require nonoperative treatment, unless the fragment is loose, causing locking, catching, pain, limping and limited range of motion.^6,8–10^ The symptoms were observed in our patient four years after the onset of LCPD. For these symptomatic patients, there are basically four treatment options: OCD fragment fixation, osteochondral autograft transfer (OAT), fresh osteochondral allograft and OCD fragment arthroscopic resection.^14–17^

Lamplot et al,^9^ in a study of 64 patients treated with hip preservation surgery for LCPD, reported that the 7 patients (7 hips) who underwent surgical hip dislocation and ORIF of femoral head OCD had significant improvement in internal rotation in flexion at final follow-up. The authors observed radiographic healing without evidence of implant failure and no progression of osteoarthritis. All 7 patients also reported marked clinical improvement with resolution of pain and mechanical symptoms at final follow-up.

Surgical hip dislocation has been used to address both intra-articular and extra-articular abnormalities around the hip joint as it is capable of addressing residual deformities of the proximal part of the femur in patients with healed LCPD.^8–12^

In this report, the patient underwent a surgical dislocation approach as described by Ganz et al,^11^ who developed the technique after remarkable understanding of the blood supply to the femoral head and the ability to safely dislocate the hip. The surgical hip dislocation approach permitted assessment and treatment of the OCD lesion with reduction of the osteochondral fragment, as described by Lamplot et al.^9^ A relative neck lengthening is also possible by means of distalization of the trochanteric flap, providing improvement in hip biomechanics, as this last restored both abductor tension and abductor lever arm.

This case report has several strengths. To the best of our knowledge, this is the 8^th^ case reported in the literature performing this technique, following its description by Lamplot et al.^9^ Our results also support the hypothesis that, when possible, surgical interventions for cartilage restoration should first attempt to preserve the patient’s native articular cartilagem.^9,15,16^ Furthermore, we included an objective quality of life questionnaire (HHS) that showed a good clinical outcome at final follow-up.

A limitation of this report is related to its retrospective nature and all the biases associated with it. Also, we didn’t have the opportunity to collect a new scanogram to analyze a possible leg length discrepancy at final follow-up, which could be related to the mild improvement in his Trendelenburg Gait. MRI and CT scans at his last visit were also not available. In addition, the follow-up time was relatively short (one year), although the patient had already reached a healed stage of the disease and showed improved outcomes.

In conclusion, the surgical hip dislocation and OCD fragment fixation technique has been found to be beneficial in many aspects. The surgery allowed simultaneous approach to the intra-articular lesion, as well as it restored the proper hip bio-mechanics, decreasing pain, improving range of motion and joint congruency, while preserving the original cartilage of the patient’s hip. It also gives the surgeon the opportunity to assess hip stability, the presence of femoroacetabular impingement and labral tears, allowing a wide variety of options for the treatment of the healed LCPD.
